# Patient-Reported Outcome Measurements in Patients with COPD-Obstructive Sleep Apnea Overlap Syndrome: Time for Action?

**DOI:** 10.3390/jpm12121951

**Published:** 2022-11-24

**Authors:** Andriana I Papaioannou, Evangelia Fouka, Evangelia Nena, Petros Bakakos, Paschalis Steiropoulos

**Affiliations:** 1 1st Department of Pulmonary Medicine and Intensive Care Unit, Medical School, National and Kapodistrian University of Athens, 15772 Athens, Greece; 2 Pulmonary Department, General Hospital G. Papanikolaou, Aristotle University of Thessaloniki, 54124 Thessaloniki, Greece; 3 Laboratory of Social Medicine, Medical School, Democritus University of Thrace, 68100 Alexandroupolis, Greece; 4 Department of Pneumonology, Medical School, Democritus University of Thrace, 68100 Alexandroupolis, Greece

**Keywords:** chronic obstructive pulmonary disease, obstructive sleep apnea, overlap syndrome, patient-reported outcome, patient-reported outcome measurements

## Abstract

Chronic obstructive pulmonary disease (COPD) and obstructive sleep apnea syndrome (OSA) are common conditions that often coexist [Overlap syndrome (OS)]. OS has important implications in the diagnosis, treatment, and patient outcome of both disorders. Patient-reported outcomes (PROs) are essential to evaluate symptoms, impact of symptoms on activities of daily living, and treatment response. The present review aims to display the potential usefulness of PROs measurements (PROMs) regarding the initial evaluation and treatment of both conditions (COPD and OSA) in OS patients. More specifically, we review PROMs regarding symptoms, mental health indices and health-related quality of life in patients with OS. These PROMs have the potential to add value to clinical research and daily practice in certain aspects that are important to patients.

## 1. Introduction

Chronic obstructive pulmonary disease (COPD) is the third leading cause of death worldwide and a crucial public health problem characterized by persistent respiratory symptoms and airflow limitation due to airway and alveolar abnormalities, usually provoked by significant exposure to noxious particles or gases [1]. The global prevalence of COPD is estimated at 3.9% of the population [2]. At the same time, obstructive sleep apnea (OSA), which is characterized by repetitive episodes of upper airway closure during sleep, is also a frequent disorder that affects 9–38% of the adult population, while its prevalence increases with age [3]. Comorbidities of OSA include cardiovascular disease, hypertension, cognitive impairment, and diabetes, which counteract and are often aggravated by OSA [4].

Since both disorders are frequent, they often coexist; therefore, the term “overlap syndrome” was subsequently introduced to describe the co-existence of both COPD and OSA in a single patient [5]. Prevalence of overlap syndrome (OS) in the general population is between 1–3.6%, ranging from 3 to 66% in COPD patients and from 7 to 55% in OSA patients [6,7,8,9]. It has been reported that patients with OS seem to have a worse prognosis than patients with either COPD or OSA alone. OS presents a higher risk of death and hospitalization because of COPD exacerbations, increased all-cause mortality, and increased risk of cardiovascular and metabolic comorbidities such as cardiac dysrhythmias, pulmonary hypertension, and right heart failure [4,10,11,12,13,14,15,16,17,18].

A patient-reported outcome (PRO) is a health outcome directly reported by the patient who experiences it. Several methods for assessing PROs exist, such as questionnaires and scales that are used to understand a patient’s state and to evaluate a treatment’s efficacy or effectiveness [19]. Patients can either complete these questionnaires and scales alone or answers can be provided through patient interviews with the precondition that the interviewer records the patients’ views and does not use the responses to make a professional judgment of his/her status. Patient involvement is the main reason why PROs are used to assess the patient’s perspective rather than measures of clinical evaluation [20]. PRO questionnaires should be designed to measure specific characteristics; in cases where a single questionnaire is about to measure multiple characteristics, separate compartments should be made to assess each one of them, while the results could be presented either as a single or as multiple scores [21].

PROs are designed to be used in any disease population (generic PROs) or are condition-targeted (used in specific medical conditions). Usually, they measure symptoms (impairments), function (disability), and general health status and health perceptions (health-related quality of life-HRQoL) [20]. In the case of COPD, several PROs have been used for the assessment of symptoms (dyspnea, cough, wheeze, chest tightness etc.), functioning (activity of daily living, energy, work), and HRQoL (usually multidimensional questionnaires assessing a combination of information about disabilities which are related to a patient’s health status) [22]. On the other hand, in OSA, PROs questionnaires mainly include symptom scales (mainly sleepiness and fatigue), functioning (assessing the degree of difficulty of being active due to fatigue or being sleepy), and quality of life (QoL) (related to either disease related or treatment impairment of activities, feelings and behaviors) [23]. In this regard, several PROs measurements (PROMs) could be used in both initial evaluation and response to treatment in patients with OS.

To assess the usefulness of PROMs in patients with the coexistence of COPD and OSA, we must focus on the specific characteristics of these patients regarding the symptoms and disability caused by both diseases and the effect of treating each condition separately. The present review aims to analyze the potential usefulness of PROMs in patients with OS regarding the initial evaluation and treatment evaluation for both COPD and OSA.

## 2. Search Strategy

Pubmed, EMBASE, and Google Scholar were searched using combinations of the following keywords: patient reported outcomes and chronic obstructive pulmonary disease, patient reported outcomes and obstructive sleep apnea, obstructive sleep apnea and overlap syndrome, patient reported outcomes and chronic obstructive pulmonary disease and sleep apnea. Only studies written in English and published up to June 2022 were included. Some overlapping articles were not included.

## 3. PROMs for the Evaluation of Symptoms in Patients with OS

Patients with sleep apnea mainly complain about symptoms such as sleepiness, fatigue, morning headache, or sore throat, while patients with COPD mainly complain about dyspnea, wheezing and chest tightness, and reduced activity [24]. All these symptoms also influence HRQoL [25]. The following PROMs have been developed to evaluate each of the symptoms mentioned above.

Epworth Sleepiness Scale (ESS) is the main scale for evaluating sleepiness. ESS is a self-administered questionnaire containing eight questions. Each question is scored on a 4-point scale (0–3) and evaluates the chance that the patient falls asleep while engaged in eight different usual everyday activities. The ESS score ranges from 0 to 24, with higher scores indicating a higher sleepiness [26]. ESS has been widely used in OSA patients and has been documented to correlate to disease severity since it is associated with the frequency of apneas [27]. Furthermore, OSA treatment with CPAP results in the significant decrease in ESS scores, indicating that ESS can be used to evaluate treatment success in OSA patients [28]. OS is known to cause more significant oxygen disturbances than either OSA or COPD alone [29,30], resulting in significant reductions in sleep efficiency, oxygen desaturations, increased arousal index, and daytime sleepiness [29]. In this regard, ESS has been also used to evaluate the possible presence of sleep-disordered breathing in COPD patients, and it has been found that patients with OS express a higher value of ESS compared with patients with COPD only [8,31,32]. However, a recent study has shown that ESS cannot predict the co-existence of OSA in COPD patients [33] and should be used with caution [34].

Sleepiness and fatigue in OSA patients can be also evaluated using the Functional Outcomes of Sleep Questionnaire (FOSQ) [35]. It comprises 30 items on a 4-point Likert scale assessing effects of being sleepy or tired on functional performance in five domains of health (activity level, general productivity, vigilance, intimate relationships, and social outcome). The total score ranges between 5 and 20, and a lower score indicates worse HRQoL [35]. In patients with OSA, FOSQ seems to be related to disease severity according to the apnea-hypopnea index (AHΙ) [36] and has been also used for evaluating the effects of treatment with CPAP showing a significant reduction after therapy, especially in patients with good adherence [37]. FOSQ has been used to evaluate the effect of nocturnal desaturation in a population of COPD patients but scores did not seem to differ between patients who experienced nocturnal desaturation and those who did not [38]. Although FOSQ has not been studied in patients with OS, we suggest that it could be tested as potential tool to provide information regarding the severity of obstructive sleep apnea and treatment response.

Baseline/Transition Dyspnea Indexes (BDI/TDI) have been developed to evaluate chronic dyspnea and its variations. The BDI is a baseline measure of chronic dyspnea that evaluates the presence of dyspnea in a single state based on three components that evoke dyspnea in activities of daily living, while the TDI quantifies the change from baseline [39]. In COPD, this measurement has been widely used and has been found to correlate with exercise capacity (measured by the 12 minutes walking distance test) [39], Forced expiratory volume in one second (FEV_1_), symptom and activity components on SGRQ, and physician’s global evaluation [40,41]. Lately, BDI/TDI has been also used to evaluate the efficacy of inhalation treatment in COPD [42,43]. In patients with sleep apnea, BDI/TDI has been evaluated before and after CPAP treatment and an increase in TDI score in both one and three months post-treatment has been reported [44]. Again, there are no studies evaluating BDI/TDI in patients with OS; however, its associations with both lung function impairment and its previously reported improvement after CPAP treatment also make this PROM a potential candidate to be used in future studies for the evaluation and follow up of patients with OS.

The Exacerbations of Chronic Obstructive Pulmonary Disease Tool-Patient-Reported Outcomes (EXACT-PRO) is a tool designed to measure directly symptoms of a COPD exacerbation reported by patients and to standardize the assessment of the patient’s condition to capture this dynamic process [45]. It has been designed to assess the frequency, severity, and duration of COPD exacerbations [45] and has been widely used in clinical trials for the evaluation of treatment efficacy on exacerbation prevention or treatment [46,47]. COPD patients with sleep disturbances have been found to be more prone to exacerbations [48], while patients with documented OS had more frequent COPD exacerbations and more hospital admissions due to COPD exacerbation compared with patients with COPD only [10]. Significantly, OSA treatment with CPAP in patients with OS results in fewer exacerbations than in untreated patients [10]. Although to our knowledge there are no studies evaluating the use of EXACT-PRO in patients with OS, this tool might be useful for the evaluation of exacerbation frequency and severity as well as treatment response in this patient population and should be tested in future studies. An overview of PROMs regarding symptoms are presented in Table 1.

## 4. PROMs for the Evaluation of HRQoL in Patients with OS

One of the main QoL questionnaires used for both COPD and OSA patients is the 36-Item Short Form Survey (SF-36), a self-completed questionnaire comprising 36 questions covering eight different health domains [49,50]. These domains evaluate limitations in physical, social, or usual role activities due to physical or emotional problems, bodily pain, psychological distress and well-being, energy and fatigue, and general health perceptions. The minimal important difference in this questionnaire is 3 points. The SF-36 is often used as a mean to evaluate QoL and has been widely used in both COPD [51,52] and OSA studies [53,54,55,56,57,58]. In the case of COPD, SF-36 seems to be related to disease severity and symptoms (according to the COPD assessment test, CAT) [59,60], while cases of OSA have shown that sleep apnea has substantial adverse effects on subjective health while OSA treatment results in improvements [53,54,55,56,57,58]. Although no studies have evaluated the use of SF-36 in OS, the proven usefulness of this material in COPD and OSA makes it also a candidate for this group of patients.

The 12-Item Short Form Survey (SF-12) is a self-reported outcome measure that assesses health’s impact on an individual’s everyday life and is often used to evaluate QoL [61]. The SF-12 was developed as a shortened version of SF-36 and was created to reduce the response burden [62]. SF-12 uses the exact eight domains with SF-36 [62] and has been shown to provide similar scores [63]. Although it has been designed as a general measure of health in the general population, it has been evaluated in patients with OSA and has been shown to reflect improvements of HRQoL after CPAP treatment [64,65,66]. The SF-12 tool has been also used in COPD showing a poorer HRQoL in this group compared with non-COPD patients [67], while it has also been proved to be affected by disease severity and symptoms [68].

The primary and most used questionnaire which evaluates HRQoL in patients with respiratory diseases is the Saint George Respiratory Questionnaire (SGRQ). This questionnaire has been designed as an instrument for evaluating the impact of obstructive airways disease on the patient’s overall health, daily life, and perceived well-being [69]. The questionnaire consists of 50 items divided in two parts, evaluating frequency and severity of symptoms, activities of everyday living influenced by respiratory symptoms, impact of disease on social functioning, and psychological disturbances. In COPD, significant correlations between SGRQ scores and lung function (FEV1), symptoms, exercise capacity, and exacerbation frequency have been described [70,71]. To our knowledge, this questionnaire has not been used to date for the evaluation of disease severity or treatment response in OSA. However, a recent study has shown a significant improvement in the scores of patients who participated in a pulmonary rehabilitation program providing evidence that this questionnaire might be helpful in the assessment of the result of a therapeutic intervention in this group [72]. SGRQ has been used in patients with OS, and it has been found that these subjects have higher scores (indicating worse HRQoL) compared with patients with COPD only [31,73].

The Functional Limitations Profile (FLP) is a 136-item instrument that assesses difficulties in physical, psychological, and social functioning. It focuses on the behaviors and feelings that people report as describing themselves at the moment of the interview and includes 12 different categories of functioning and well-being, including (1) ambulation, (2) body care and movement, (3) mobility, (4) household management, (5) recreation and pastimes, (6) social interaction, (7) emotional behavior, (8) alertness behavior, (9) sleep and rest, (10) eating, (11) communication, and (12) work. The FLP has been used in patients with sleep apnea and was able to detect pre-treatment decrements in subjects’ QoL, as well as substantial improvements following treatment with CPAP [54]. This tool has not been evaluated in COPD, however, given its ability to detect QoL impairment in OSA and improvements after treatment, this questionnaire could possibly be used in patients with OS.

Euro-Quality of Life-5 Dimensions (EQ-5D) questionnaire is a standardized measure of HRQoL that assesses health status in terms of five dimensions of health. It is considered a ‘generic’ questionnaire since these dimensions are not specific to any patient group or health condition, and it is considered as a PROM because patients can complete the questionnaire by themselves to provide information on their health status [74]. The questionnaire has been used in patients with sleep apnea but failed to show significant improvements with therapy, presumably because it failed to measure the aspects of QoL related to severe sleep fragmentation and daytime sleepiness [54].

The Pittsburgh Sleep Quality Index (PSQI) is a self-rated questionnaire developed to assess sleep quality in sleep disorders, as well as disturbed sleep, in other conditions such as mood disorders or pain syndromes. It consists of nineteen questions on a 4-point Likert scale (0–3) and covers seven domains: subjective sleep quality, sleep latency, sleep duration, sleep efficiency, sleep disturbance, use of sleeping medication, and daytime dysfunction. The sum of scores for these seven components yields one global score. The global score is the sum of all domain items and ranges from 0 to 21. The cutoff for abnormal sleep is >5, and worse sleep quality is associated with higher scores [75]. The PSQI has been used in patients with OSA giving data on poor sleep quality related to cardiovascular comorbidities, daytime sleepiness, and nocturnal desaturation [76]. In a study including patients with COPD, PSQI scores were worse in patients with lower diffusion capacity [77], while higher scores correlated to exacerbation frequency [78] and increased symptoms according to CAT [79]. PSQI has been also evaluated in patients with OS, showing no difference compared with patients with COPD only [80].

The Sleep Apnea Quality of Life Index (SAQLI) questionnaire was developed to measure QoL in patients with OSA. It consists of a 7-point scale from “all the time” to “not at all” and evaluates daily functioning, social interactions, emotional functioning, symptoms, and impact of treatment side-effects on QoL [81]. The tool showed impaired QoL in OSA patients compared with controls, although this impairment was not associated to disease severity [25]. Also, it was found to detect differences in QoL following CPAP treatment in patients with severe and moderate OSA but not in those with mild disease [82]. SAQLI has been used in stable hypercapnic COPD patients showing improvement in their daily functioning after high-intensity non-invasive positive pressure ventilation [83]. In this study the presence of sleep apnea was excluded; however, the design of SAQLI is such that it is worth to be evaluated in patients with OS.

The Sleep Quality Scale (SQS) has been developed to be used as an all-inclusive assessment tool for evaluating sleep quality in various patient and research populations. It consists of six domains of sleep quality: daytime symptoms, restoration after sleep, problems initiating and maintaining sleep, difficulty waking, and sleep satisfaction. The scoring ranges from 0 to 84, with higher scores denoting more acute sleep problems [84]. It has been previously used in patients with sleep apnea showing higher scores compared with control subjects [85]. SQS has been evaluated neither in COPD patients nor in patients with OS; however, its developmental methodology, together with the observation that patients with OS may have worse sleep quality than those with OSA or COPD alone [6], allows the hypothesis that it is a PROM which could be of value in these patient populations.

The COPD and Asthma Sleep Impact Scale (CASIS) is a 15-item questionnaire that assesses a combination of sleep impairment aspects with respiratory disease and breathing problems. The response options range from 1 = never to 5 = very often, and several items are scored reversely. The item scores were summed together to arrive at a total raw score [86]. CASIS has been developed to evaluate sleep quality in patients with obstructive lung diseases. Ιn COPD studies, CASIS has shown a greater impairment of sleep in more symptomatic patients and in patients with more severe obstruction [87,88]; however, it has not been used in patients with OSA. Given the specificity of this questionnaire in obstructive lung diseases, it also may be considered a candidate to be evaluated in patients with OS. An overview of PROMs regarding HRQoL are presented in Table 2.

## 5. PROMs for the Evaluation of Mental Health in Patients with OS

The Beck’s Anxiety Inventory (BAI) has been developed as a self-report measure of anxiety. It includes 21 items scoring from 0 to 3, and the total score is calculated by adding the score of each item [89]. The Beck’s depression inventory (BDI) is a 21-item, self-report rating inventory that measures characteristic attitudes and symptoms of depression [90]. It also includes 21 items that rate symptoms of depression such as hopelessness and irritability, cognitions such as guilt or feelings of being punished, and physical symptoms such as fatigue, weight loss, and lack of interest for sexual activity. BAI and BDI have been evaluated in patients with OSA showing increased levels of anxiety and depression compared with control subjects [91]. Similarly, both items have been used in COPD and have shown that both anxiety and depression scores are associated with symptom severity [92], poor HRQoL [93], and exacerbation frequency [94]. To our knowledge, these questionnaires have not been evaluated in patients with OS; however, they might be useful as screening tools for the recognition of anxiety and depression in this group of patients.

The Hospital Anxiety and Depression Scale (HADS) has been developed to evaluate the levels of anxiety and depression that an individual with physical health problems is experiencing. Thus, the item has been developed to avoid reliance on aspects of these conditions that are also common somatic symptoms of illness, such as fatigue and insomnia or hypersomnia. It is a fourteen-item questionnaire in which seven items refer to anxiety and seven to depression [95]. In OSA patients, it has been found that higher scores are associated with disease severity [96] and scores were ameliorated after CPAP treatment [97]. In COPD, HADS score can diagnose the presence of depression [98], while it was found to be associated with disease severity [99] and exacerbation frequency [100]. HADS has also been used in patients with OS that had higher scores compared with patients with COPD alone [101]. An overview of PROMs regarding mental health are presented in Table 3.

**Table 1:** PROMs related to symptoms.

Tool	Assessment	MCID (units)	COPD	OSA	Overlap Syndrome
ESS	Daytime sleepiness [26]	2 [102]	Moderate efficacy in prediction of OSA [8,31,33]	Association with apnea frequency [27] Evaluation of long-term CPAP treatment efficacy [28]	Worse sleep quality compared to COPD alone [6,31]
FOSQ	Effects of daytime sleepiness on functional performance [35]	1 [37]	Assessment of sleep quality and daytime function (ns differences in desaturators versus non-desaturators)	Relation to disease severity (AHI) [36] Evaluation of CPAP treatment efficacy [37]	N/A
BDI/TDI	BDI: dyspnea severity in a single state, TDI: changes in dyspnea severity from baseline (as established by the BDI) [39]	BDI: N/A TDI: ≥1 [40]	Correlation with exercise capacity [39], lung function [39,40], symptom and activity components of SGRQ [40,41] and physician’s global evaluation [40,41] Evaluation of inhalation treatment efficacy [42,43]	Increases in TDI after treatment [44]	N/A
EXACT-PRO	Symptomatic manifestations of COPD exacerbations [45]	0.92 [47]	Detection of COPD exacerbations and evaluation of their severity and treatment efficacy [46,47]	N/A	N/A

Abbreviations: BDI/TDI: Baseline/Transition Dyspnea Indexes, COPD: Chronic Obstructive Pulmonary Disease, CPAP: Continuous Positive Airway Pressure, ESS: Epworth Sleepiness Scale, EXACT-PRO: Exacerbations of Chronic Obstructive Pulmonary Disease Tool-Patient Reported Outcomes, FOSQ: Functional Outcomes of Sleep Questionnaire, MCID: Minimal clinically important difference, OSA: Obstructive Sleep Apnea, PROMs: Patient reported outcomes measures, SGRQ: Saint George Respiratory Questionnaire.

**Table 2:** PROMs related to Health-related Quality of Life.

Tool	Assessment	MCID (Units)	COPD	OSA	Overlap Syndrome
SF-36	QoL impairment due to physical or emotional problems [49,50]	3 [49]	Moderate-good correlations of all components to disease severity and symptoms (CAT) [59,60]	Lower scores compared to general population [55] Significant correlations with sleepiness due to sleep disruptions [56,57,58] Improvements after CPAP therapy [53,54]	N/A
SF-12	Health impact on individual’s everyday life [54]	N/A	Lower scores in the physical subscale compared to non-COPD patients [67] Worse scores in both physical and mental components with increased symptoms and disease severity [68]	Improvements in HRQoL after CPAP treatment [64,65,66].	N/A
SGRG	Impact of obstructive airway diseases on overall health, daily life and perceived well-being [69]	4 [69]	Significant correlations with lung function (FEV_1_), symptoms, exercise capacity and exacerbation frequency [70,71]	Significant improvements in QoL (SGRQ) in patients who received PR plus CPAP compared to CPAP therapy alone [72]	Worse QoL compared to COPD only [31,73]
FLP	Functional limitations in physical and psychosocial well-being at the moment of the interview (https://doi.org/10.1007/978–94–007–0753–5_1100)	N/A	N/A	Pre-treatment decrements and substantial improvements in QoL after CPAP treatment [54]	N/A
EQ-5D	Generic QoL status, in terms of mobility, self-care, usual activities, pain/discomfort, and anxiety/depression [74]	N/A	N/A	No improvements in HRQoL after CPAP therapy [54]	N/A
PSQI	Sleep quality due to sleep disturbancies over a 1-month interval [75]	≥5 [75]	Lower scores in patients with reduced DL_CO_ [77] Higher scores correlated to exacerbation frequency [78] and increased symptoms (CAT) [79]	Higher scores associated with CVD comorbidities, daytime sleepiness (ESS) and nocturnal desaturation [76]	No difference in sleep quality compared to COPD only [80]
SAQLI	QoL in terms of daily functioning, social interaction, emotional functioning and symptoms [81]	N/A	Improvements in daily functioning component after high-intensity NIV therapy in stable hypercapnic COPD [83]	Impaired QoL compared to controls [25]. Long-term improvements after CPAP treatment in severe and moderate OSA [82]	N/A
SQS	Sleep quality in various populations [84]	N/A	N/A	Higher scores compared to controls [85]	N/A
CASIS	Sleep impairment associated with respiratory disease and breathing problems [86]	N/A	Greater sleep impairment in more symptomatic patients (CAT) and more severe disease (spirometry-based) [87,88]	N/A	N/A

Abbreviations: CASIS: COPD and Asthma Sleep Impact Scale, CAT: COPD Assessment Test, COPD: Chronic Obstructive Pulmonary Disease, CPAP: Continuous Positive Airway Pressure, EQ-5D: Euro QoL-5 Dimension, ESS: Epworth Sleepiness Scale, FLP: Functional Limitations Profile, HRQoL: Health-related Quality of Life, MCID: Minimal clinically important difference, NIV: Non-invasive Ventilation, OSA: Obstructive Sleep Apnea, PR: Pulmonary Rehabilitation, PROMs: Patient reported outcomes measures, PSQI: Pittsburgh Scale Quality Index, SAQLI: Sleep Apnea Quality of Life Index, SF-36: Short Form Survey 36, SF-12: Short Form Survey 12, SGRQ: Saint George Respiratory Questionnaire, SQS: Sleep Quality Scale, QoL: Quality of Life.

**Table 3:** PROMs related to mental health.

Tool	Assessment	MCID (Units)	COPD	OSA	Overlap Syndrome
BAI/BDI	BAI: measure of anxiety [89] BDI: measure of depression symptoms and severity [90]	N/A for BDI (5 for BDI-II) [103]	Associations with symptom severity [92], poor HRQoL [93] and exacerbation frequency [94]	Increased levels of anxiety and depression compared to controls [91]	N/A
HADS	Levels of anxiety and depression in a general medical population [95]	1.5 in COPD [104], 8 in OSA [96]	Diagnostic of depression [98] Associations with disease severity [99] and exacerbation frequency [100].	Higher scores in more severe disease [96] Improvements after CPAP treatment [97]	Higher scores compared to COPD alone [101]

Abbreviations: BAI: Becks anxiety inventory, BDI: Becks depression inventory, COPD: Chronic Obstructive Pulmonary Disease, CPAP: Continuous Positive Airway Pressure HADS: Hospital Anxiety and Depression Scale, HRQoL: Health-related Quality of Life, MCID: Minimal clinically important difference, OSA: Obstructive Sleep Apnea, PROMs: Patient reported outcomes measures.

## 6. Conclusions

Several PROMs are suitable for evaluating symptom severity, impact of the disease, and impairment of daily functioning and QoL in patients with either OSA or COPD. Since OS is characterized by a combination of symptoms related to OSA and COPD, PROMs that have not been designed to be disease-specific seem to be more useful for the evaluation of these patients. However, the use of each PROM in this specific population needs be evaluated to prove its clinical significance related to disease severity, outcome, and treatment response. However, one has to admit that the use of disease-specific PROMs by non-experts includes the risk of misdiagnosis or inadequate evaluation of treatment benefits.

PROMs are especially meaningful when evaluating disease burden, both in OSA and COPD, as they include evaluation of symptoms and activities that patients highly value. However, regarding OS, none of the identified PROMs has been fully validated, and many validation studies were of insufficient quality. Moreover, the lack of established content validity for most of the PROMs measuring health status is problematic for a patient-centered approach, as the items of these PROMs might not address the issues patients with OS consider relevant or most important, and the use of generic PROMs will by definition contain questions less relevant for the specific disease. Therefore, in order to differentiate OS from OSA and COPD populations, individual questions of some PROMs might be more suitable for alerting the treating physician to the most critical problems of these patients; for example, rather than relying on composite scores of the domains, the “daytime sleepiness” domain of the QSQ would be suitable.

The ESS questionnaire, measuring only sleepiness, is the most widely used PROM in OS. However, the quality of this PROM is moderate at best. In our opinion, the only PROMs with good content validity are three OSA-related quality-of-life PROMs: the PSQI, the QSQ, and the SAQLI questionnaires. Therefore, we consider these PROMs the most suitable for a patient-centered approach to health status and all three of them potentially suitable for outcomes measurements. Even though there is not enough evidence to fully judge the quality of these PROMs, they can potentially add value to outcome measurement or clinical practice when they are interpreted with caution in individual patients. Unanswered questions still exist regarding the identification of distinct symptom-based phenotypes in the context of disease diversity in OS and their associations with clinically meaningful outcomes.

On the other hand, the fact that PROMs provide direct assessment of the patients’ needs and perspectives allows a more objective evaluation of the medical condition and disease impact on their everyday life, a fact that makes the use of these tools important. What might be challenging on the use of several PROMs would be their potential ability to suspect the presence of OS in a patient with diagnosed COPD, even in the primary care setting, in order to be referred to special sleep centers, resulting in less undiagnosed conditions and less unnecessary referrals. Undoubtedly, sleep in patients with COPD might be impaired by several other factors such as nighttime symptoms, dyspnea, or cardiovascular comorbidities and not necessarily to the presence of sleep apnea. Disease-specific PROMs in these populations might be proved more relevant to differentiate these conditions; however, this has to be evaluated in the future. The use of digitized PROs, or electronic patient-reported outcomes (ePROs), which have been on the rise in the health research setting during the latest years, could be also help in this direction.

## Data Availability

Not applicable.
